# Medical Association of Georgia

**Published:** 1883-05

**Authors:** 


### MEDICAL ASSOCIATION OF GEORGIA,

### IN SESSION AT ATHENS, 18TH AND 19TH OF APRIL.

The Association was called to order by the retiring President, Dr. Wm. F. Holt, of Macon, Dr. Campbell, the Secretary being absent.

Dr. James A. Gray, of Atlanta, was appointed Secretary pro tem.

Dr. John Gerdine, of Athens, made the address of welcome, which was responded to by Dr. James B. Baird.

The following officers were elected: President, Dr. A. W. Calnoun, of Atlanta; First Vice-President, Dr. R. J. Nunn, of Savannah; Second Vice-President, Dr. M. P. Deadwyler, of Elberton; Secretary, Dr. James A. Gray, of Atlanta.

Dr. J. S. Todd, of Atlanta. and Dr. A. W. Griggs, of West Point, were elected Censors.

The next meeting of the Association will be held in Macon.

The most important act of this session was the appointment of a committee of nine to memorialize the Legislature of the State to take some steps for the regulation of dissecting.

As the law now stands in this regard, it presents one of the most inviting subjects for burlesque that a humorist could desire. It is a little remarkable how it has so long eluded the vicious eyes of the *Texas Siftings* and the *Georgia Major*.

The idea of a great sovereign State enacting laws on the one hand to punish ignorance and malpractice in medical men, and per contra, a statute that makes it a felony to dissect for purposes of professional education, a dead human body. It is precisely in keeping with the Irish bullism that would prompt a legislative body to declare public education compulsory, and at the same time make any man a felon, or an offender who would open a school house.

It does appear that the Atlanta *Constitution* is shadowing the truth when it refers so frequently to certain Potaphir Peagreens, who some way manage to dodge into the Legislature of Georgia.

Why did not the Association assign to the same committee the duty of looking after the sanitation of that poor abortion of a State Board of Health that still lives, a bloodless apparition, mocking the hopes of an intelligent people, and shaking its bony finger in the face of the goddess of health.

